# Cone-beam computed tomography in hypofractionated stereotactic radiotherapy for brain metastases

**DOI:** 10.1186/1748-717X-7-54

**Published:** 2012-04-01

**Authors:** Gianluca Ingrosso, Roberto Miceli, Dahlia Fedele, Elisabetta Ponti, Michaela Benassi, Rosaria Barbarino, Luana Di Murro, Emilia Giudice, Federico Santarelli, Riccardo Santoni

## Abstract

**Background:**

To assess interfraction translational and rotational setup errors, in patients treated with image-guded hypofractionated stereotactic radiotherapy, immobilized by a thermoplastic mask and a bite-block and positioned using stereotactic coordinates.

**Methods:**

37 patients with 47 brain metastases were treated with hypofractionated stererotactic radiotherapy. All patients were immobilized with a combination of a thermoplastic mask and a bite-block fixed to a stereotactic frame support. Daily cone-beam CT scans were acquired for every patient before the treatment session and were matched online with planning CT images, for 3D image registration. The mean value and standard deviation of all translational (X, Y, Z) and rotational errors (θ_x_, θy, θ_z_) were calculated for the matching results of bone matching algorithm.

**Results:**

A total of 194 CBCT scans were analyzed. Mean +/- standard deviation of translational errors (X, Y, Z) were respectively 0.5 +/- 1.6 mm (range -5.7 and 5.9 mm) in X; 0.4 +/- 2.7 mm (range -8.2 and 12.1 mm) in Y; 0.4 +/- 1.9 mm (range -7.0 and 14 mm) in Z; median and 90th percentile were respectively within 0.5 mm and 2.4 mm in X, 0.3 mm and 3.2 mm in Y, 0.3 mm and 2.2 mm in Z. Mean +/- standard deviation of rotational errors (θ_x_, θy, θ_z_) were respectively 0.0 degrees+/- 1.3 degrees (θ_x_) (range -6.0 degrees and 3.1 degrees); -0.1 degrees +/- 1.1 degrees (θy) (range -3.0 degrees and 2.4 degrees); -0.6 degrees +/- 1.4 degrees (θ_z_) (range -5.0 degrees and 3.3 degrees). Median and 90th percentile of rotational errors were respectively within 0.1 degrees and 1.4 degrees (θ_x_), 0.0 degrees and 1.2 degrees (θy), 0.0 degrees and 0.9 degrees (θ_z_). Mean +/- SD of 3D vector was 3.1 +/- 2.1 mm (range 0.3 and 14.9 mm); median and 90th percentile of 3D vector was within 2.7 mm and 5.1 mm.

**Conclusions:**

Hypofractionated stereotactic radiotherapy have the significant limitation of uncertainty in interfraction repeatability of the patient setup; image-guided radiotherapy using cone-beam computed tomography improves the accuracy of the treatment delivery reducing set-up uncertainty, giving the possibility of 3-dimensional anatomic informations in the treatment position.

## Introduction

Brain is a common site for metastases. Stereotactic radiosurgery (SRS) and hypofractionated stereotactic radiotherapy (HSRT), due to their ability to deliver very high doses to a small volume with high tumour control rates [1], may be considered as a standard treatment for patients with brain metastases. From a biological point of view HSRT might have some advantage in comparison to SRS in terms of acute complications [2] and of tumour control rate for lesions larger than 10 cc (or more than 3 cm of diameter) [3,4]. In HSRT, daily treatment reproducibility is necessary in order to avoid geographic miss of the target; as brain metastases are not affected by internal organ motion and their position can be considered stably correlated with bony structures, patient set-up is the crucial step for the exact treatment delivery. Several noninvasive systems are used for patient immobilization, but verification of the patient position is always necessary to enhance the precision of the stereotactic treatment. Image-guided radiotherapy (IGRT) using cone-beam computed tomography (CBCT) offers the possibility of a daily precise detection and correction of translational and rotational set-up errors.

The aim of this study was to assess inter-fraction translational and rotational set-up errors, in patients treated with IGRT-HSRT immobilized by a thermoplastic mask and a bite-block and positioned using stereotactic coordinates.

## Methods and materials

Between April 2008 to September 2010, 37 patients with 47 brain metastases were treated with IGRT-HSRT at the Radiation Oncology Therapy Unit of the University of Rome, Tor Vergata. Inclusion criteria were: maximum 3 brain metastases, ≤ 3.5 cm of diameter and good performance status (Karnofsky performance status ≥ 70). Table 1 summarized patient and treatment features. Primary cancers were non-small-cell lung cancer in the majority cases (n = 17), breast cancer (n = 9), colon cancer (n = 4), melanoma (n = 3) and other sites (n = 4). The median planning target volume of the 47 metastases was 2.3 cc (mean 1.6 cc; range 0.65 cc - 14.3 cc) with a median diameter of 16.7 mm (mean 14.5 mm; range 5 mm - 35 mm).

**Table 1:** Patient and treatment characteristics

	Patients (37)	Lesions (47)	Volume
	
	**No**.	**No**.	cc
**primary tumour**			

Lung	17		

Breast	9		

Colon	4		

melanoma	3		

other sites	4		

**Planning target volume**			

Mean			1.6

Range			0.65 -14.3

**total dose and** **fractionation**			

18 Gy (3 × 6 Gy)		6	

24 Gy (4 × 6 Gy)		4	

30 Gy (2 × 15 Gy)		1	

30 Gy (5 × 6 Gy)		6	

32 Gy (4 × 8 Gy)		29	

40 Gy (5 × 8 Gy)		1	

**Figure 1:**
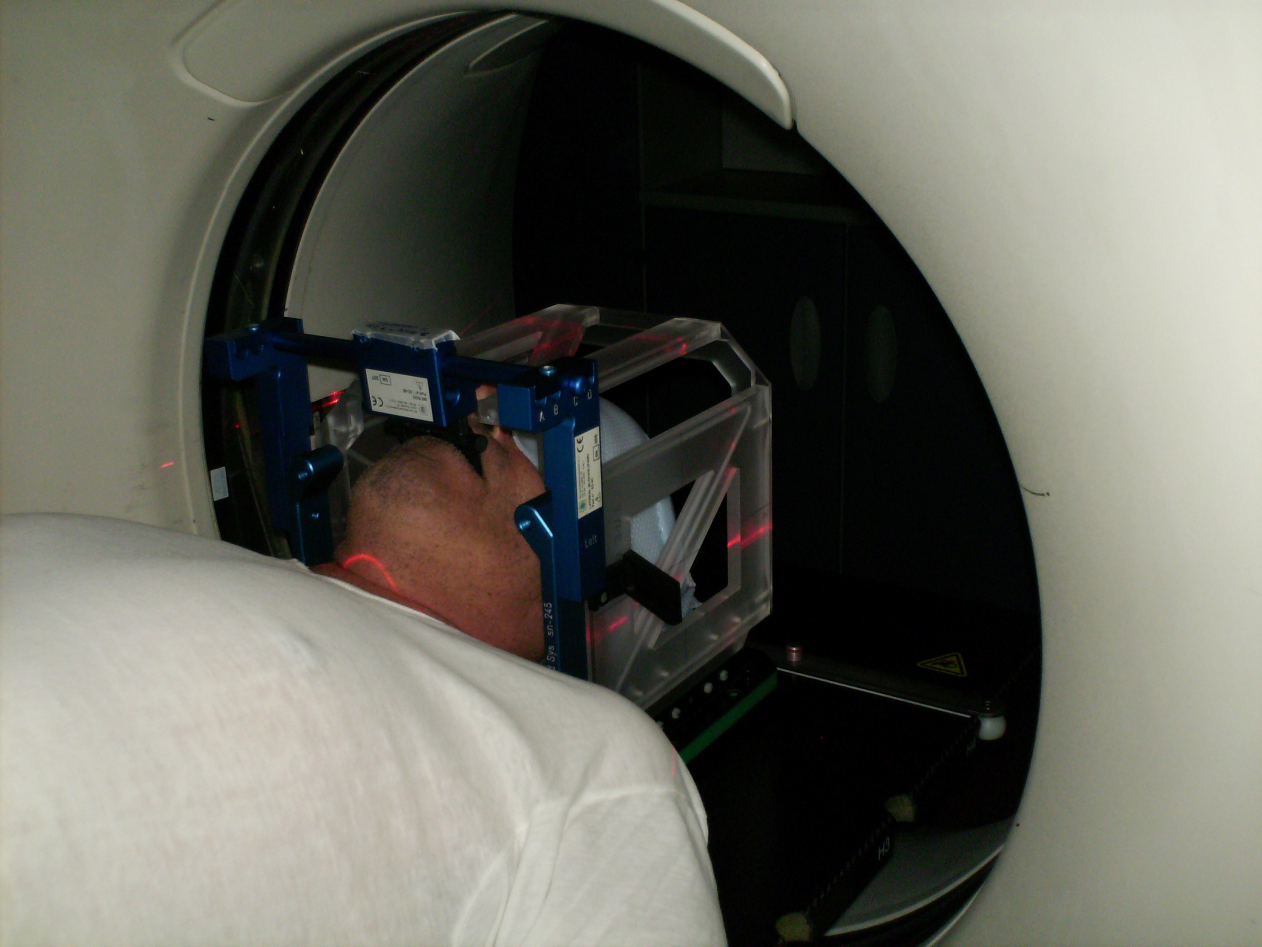
**Patient immobilized with the combination of the thermoplastic mask and the bite-block fixed to the stereotactic frame support, with the frame for stereotactic coordinate generation applied over the mask**.

All patients were immobilized with a combination of a thermoplastic mask (Head Mask R-PRT3, Klarity) and a bite-block (3DLine^®^) fixed to a stereotactic frame support (Head Frame, 3DLine^®^); the frame for stereotactic coordinate generation (Multimodality Localizer CT/MRI, 3DLine^®^) was applied over the mask (Figure 1).

A contrast-enhanced CT scan (GE LightSpeed^® ^Scanner; GE Healthcare Diagnostic Imaging, Slough, UK) with a 1.25 mm slice thickness was acquired in axial mode for planning. All patients had an MRI study that was used for image registration in the planning; CT images were transferred to Ergo stereotactic treatment planning system (Elekta 3DLine^® ^Medical System PMM Vers.1.6.3.1.) in order to contour the gross tumour volume (GTV), that consisted of the radiographically evident contrast-enhancing gross disease, and to determine the stereotactic localization of the isocenter. CT images with a marker point (isocenter) and GTV contours were transferred from Ergo to Pinnacle (Philips Medical System, Andover, MA); CT and MRI images were automatically registered on Syntegra software (Pinnacle, Philips Medical System, Andover, MA). The CT-contoured GTV was corrected on CT-MRI registration; planning target volume (PTV) and organs at risk (OARs) were delineated. The PTV was defined as the GTV plus a 3 mm isotropic margin. Treatment plans were produced on Pinnacle3 version 8.0 m (Philips Medical System, Andover, MA); multiple (5-9) no-coplanar beams (6 MV photons) were used to perform the treatment plan. The median isodose at the periphery of the PTV was 95%, the mean isodose 95.6% (range 91%-100%); for every lesion the total dose was prescribed to the isodose at the periphery of the PTV. Different fractionation schedules were used, based on tumour size and site: 3 fractions of 6 Gy (6 lesions), 4 fractions of 6 Gy (4 lesions), 5 fractions of 6 Gy (6 lesions), 2 fractions of 15 Gy (1 lesion), 4 fractions of 8 Gy (29 lesions), 5 fractions of 8 Gy (1 lesion). For every organ at risk we started from the Biologic Effective Dose (BED) definition to calculate the equivalent max dose for every fractionation scheme:

$$BED\left(n,d;\alpha /)\beta \right)=nd\left(1+\frac{d}{\alpha /)\beta }\right)$$

where *n *is the number of fractions, *d *is the fraction dose.

Patients were treated with Linac Elekta Synergy^® ^S (Elekta Oncology Systems, Crawley, UK) equipped with Beam ModulatorTM (leaf width of 0.4 cm at the isocenter) and a kilovolt (kV) imaging system capable of acquiring 3D X-ray volume images based on kV cone-beam computed tomography (CBCT). In particular CBCT has the tube and flat panel imager both mounted on retractable arms that extend from the accelerator's drum structure. The kilovoltage system is mounted in an orthogonal direction to the MV system sharing a common axis of rotation. We used the "head and neck protocol" with the following parameters: 100 kV, 36.1 mAs, nominal scan dose 0.9 mGy, FOV (Field Of View) at the isocenter 260.4 mm. All the images were stored and processed on a control work station (XVI).

For every patient, planning CT images, with OARs, PTV and marker isocenter were transferred from Pinnacle to XVI.

Daily CBCT scans were acquired for every patient before the treatment session. Projections are acquired during a single rotation of the gantry (about 113 s) and processed with Elekta XVI software (XVI, Elekta, Crawley, United Kingdom) that with a back-projection algorithm reconstructs the 3D volumetric images. Reconstruction of about 625 projections is performed on a Intel Xeon (TM) 3.06 GHz processor and it takes about 30 s to reconstruct a 410 × 264 × 410 voxel matrix with voxel dimension 1 × 1 × 1 mm^3^.

Planning CT images were matched online with the daily CBCT images using bone-matching algorithm for 3D image registration (chamfer matching).

An alignment clip-box for volumes matching was defined by the physicians. The registration was checked by a physician using a "cut" display modality. After 3D registration the XVI software calculates translational and rotational set-up errors. On-line corrections were performed before each treatment. The translational errors were corrected using the mechanical movements of the couch; the rotational errors were corrected using the knobs of the stereotactic head frame support.

If the translational and rotational errors in the first set-up exceeded respectively 6 mm and/or 3 degrees, the patient was repositioned.

The mean value and standard deviation of all translational (X, Y, Z) and rotational errors (θ_x_, θy, θ_z_) were calculated for the matching results of bone matching algorithm. It was calculated the length of the translational correction 3D vector using the formula3D vector = (X^2^+Y^2^+Z^2^)^1/2^.

To test the reproducibility of isocenter detection and position correction an intense program of quality assurance tests was performed: a home made phantom and an anthropomorphic phantom were used to assess error correction performance [5].

## Results

**Figure 2:**
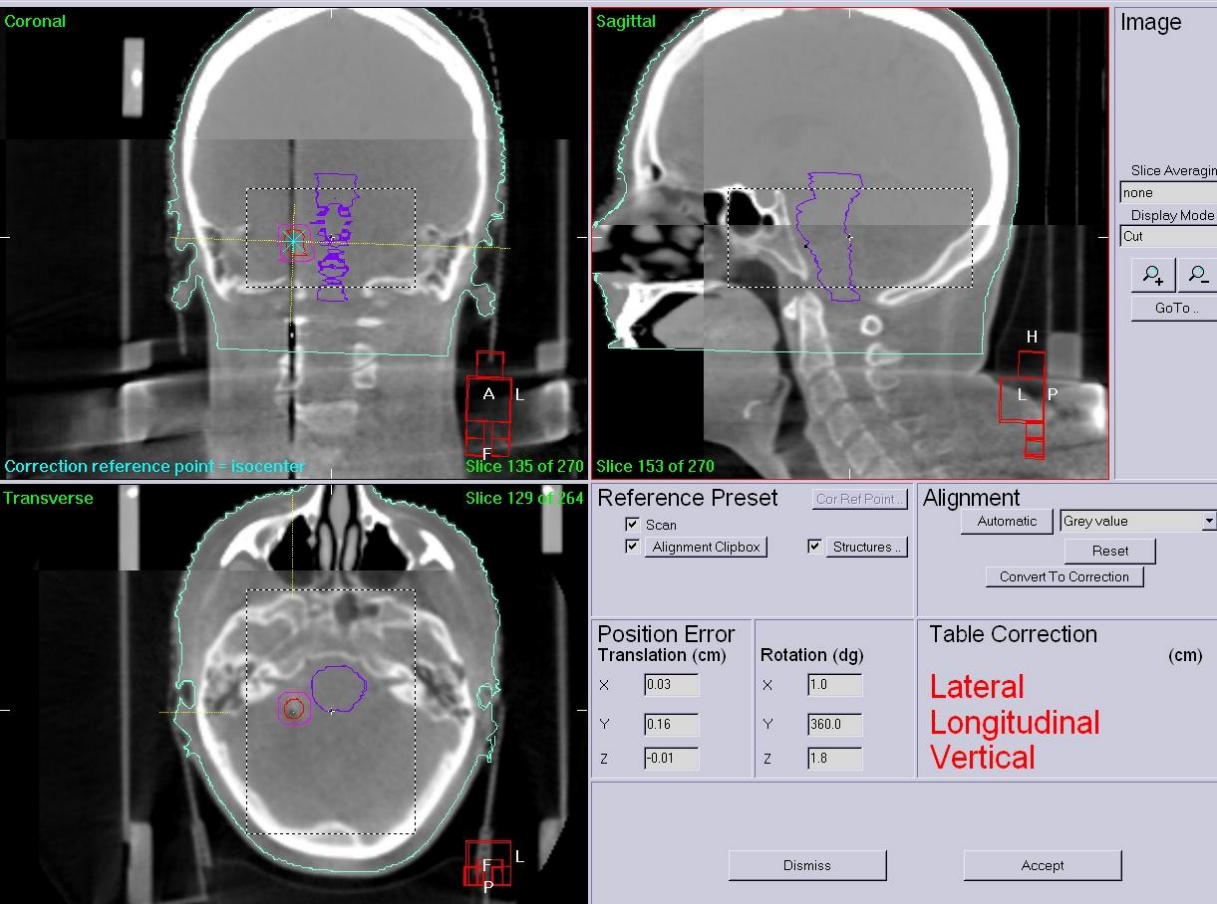
**Cone-beam computed-tomography (CBCT) matched with the planning CT using the clip-box involving skull, pneumatic sinusal structure and clivus**.

A total of 194 CBCT scans were retrospectively analyzed to evaluate the positioning errors obtained by automatic bone alignment. Before each fraction of hypofractionated stereotactic radiation therapy a CBCT was acquired with the "head and neck protocol" and a clip-box involving skull, pneumatic sinusal structure and clivus was chosen to determine the match volume (Figure 2).

For the 194 CBCT, mean, median, standard deviation (SD), 90^th ^percentile and range of set-up errors were calculated along the three major directions [lateral (X), cranio-caudal (Y) and antero-posterior (Z)] considering the totality of the data obtained for all patients and all fractions. The same parameters were evaluated for rotational set-up errors (θ_x_), (θy), (θ_z_).

The results of the corrections obtained by XVI software are listed in Table 2. Mean ± SD of translational errors (X, Y, Z) were respectively 0.5 ± 1.6 mm (X axis) (range -5.7 and 5.9 mm); 0.4 ± 2.7 mm (Y axis) (range -8.2 and 12.1 mm); 0.4 ± 1.9 mm (Z axis) (range -7.0 and 14 mm).

**Table 2:** Patient repositioning errors (194 CBCT)

		tranlations (mm)			rotations (°)		
	**x**	**y**	**z**	**3D vector**	**θ_x_**	**θ_y_**	**θ_z_**

**mean**	0.5	0.4	0.4	3.1	0	-0.1	-0.6

**SD**	1.6	2.7	1.9	2.1	1.3	1.1	1.4

**median**	0.5	0.3	0.3	2.7	0.1	0	0

**90th percentile**	2.4	3.2	2.2	5.1	1.4	1.2	0.9

**range**	-5.7; 5.9	-8.2; 12.1	-7; 14	0.3; 14.9	-6; 3.1	-3; 2.4	-5; 3.3

Median and 90^th ^percentile of translational errors were respectively within 0.5 mm and 2.4 mm (X axis), 0.3 mm and 3.2 mm (Y axis), 0.3 mm and 2.2 mm (Z axis).

Mean ± SD of rotational errors (θ_x_, θy, θ_z_) were respectively 0.0° ± 1.3° (θ_x_) (range -6.0° and 3.1° mm); -0.1° ± 1.1° (θ_y_) (range -3.0° and 2.4°); -0.6° ± 1.4° (θ_z_) (range -5.0° and 3.3°).

Median and 90^th ^percentile of rotational errors were respectively within 0.1° and 1.4° (θ_x_), 0.0° and 1.2° (θy), 0.0° and 0.9° (θ_z_). Mean ± SD of 3D vector was 3.1 ± 2.1 mm (range 0.3 and 14.9 mm); median and 90^th ^percentile of 3D vector was within 2.7 mm and 5.1 mm.

## Discussion

Stereotactic radiotherapy is a treatment modality for brain metastases to deliver very high doses to a small volume and to obtain high tumour control rates. Hypofractionated stereotactic radiotherapy has the advantage to deliver large doses - probably more effective at killing radioresistant tumours- with the radiobiological features of fractionation [6,7]; in fact, from a biological point of view it seems to be better than SRS in terms of acute complications [2] and of tumour control rate for lesions larger than 10 cc [3,4]. To avoid geographic miss of the target and to spare organs at risk from irradiation, high precision in the daily set-up is required. In stereotactic radiosurgery this precision can be achieved using an invasive rigid fixation of the skull to a stereotactic system that is placed for immobilization before the planning, and it is removed at the end of the treatment delivery. In fractionated stereotactic radiotherapy we use non invasive fixation, such as bite-block and/or a mask system, introducing a significant variation in daily set-up. The uncertainties, related to the non invasive fixation system, can result from different causes such as weight changes of the patient (weight gain for patients receiving steroids), different mask-making technique, thermoplastic mask shrinking [8].

The combination of two relocatable immobilization devices, such as thermoplastic mask and bite-block, is more effective for head treatment-position reproducibility. Recently, Masi et al. evaluated set-up errors measured by a CBCT for patients immobilized by a thermoplastic mask and a bite-block; when patients were completely repositioned, the set-up corrections were significantly larger than those measured when patients were left immobilized, and set-up errors measured with mask alone were larger than those obtained with a mask and a bite-block, but not with statistically significant difference. In the analysis of 131 CBCT for 57 patients receiving SRT, they obtained an overall mean and standard deviation for the 3D vector of 3.0 ± 1.4 mm and a maximum standard deviation of 2.4 mm that was observed along the cranio-caudal direction [9].

In the study of Boda-Heggemann et al. [10] the accuracy of two different mask systems (rigid mask vs thermoplastic mask), using CBCT three-dimensional matching, was compared; the mean module of 3D displacement vector was 3.1 mm ± 1.5 mm for patients immobilized with rigid mask and 4.7 ± 1.7 mm for those immobilized with the thermoplastic mask; Guckenberger et al. measured a mean three-dimensional set-up error of 4.0 ± 2.1 mm according to the bony anatomy of the skull, in 18 patients treated with image-guided stereotactic radiotherapy using kv-CBCT. All these patients underwent a CBCT and a conventional CT after injection of i.v. contrast, just before the treatment session. Set-up errors using automatic bone registration (CBCT) and manual soft tissue registration of brain metastases (conventional CT) were compared, demonstrating a significant correlation between the two types of registration (*r *≥ 0.88) and thus the great accuracy of daily repositioning with image-guidance based on the bony anatomy of the skull that can be used as a surrogate for the actual target position [11].

In the present study we evaluated set-up accuracy using automatic image registration between on-board kV CBCT and planning CT scan, based on a clipbox involving the base of the skull; a system based on mask and bite-block immobilization was used. A total of 194 CBCT have been examined. We obtained an overall mean and standard deviation for the 3D vector of 3.1 ± 2.1 mm, that is comparable with data obtained by other authors, with a range of the 3D vector between 0.3 mm and 14.9 mm; we registered the widest range of set-up errors in the cranio-caudal direction (between -7 mm and 14 mm). The analysis of daily repositioning errors shows that errors related to mask-making technique and patient compliance have to be considered for a stereotactic treatment; in our series in 17 cases we had a translational error exceeding 6 mm and/or a rotational error > 3 degrees and we repeated all the set-up procedure assuming that this difference was attributable to the wrong position of the head into the mask, most of all due to the movements of the joint between the occipital bone and the first cervical vertebra, or to radiation therapist random errors.

The accuracy of automatic 3D-3D matching for evaluation of patient set-up is better than manual 2D-2D matching; for Elekta XVI automatic registration, phantom studies have shown errors less than 0.5 mm [12,13]. The fractionated stereotactic radiotherapy needs IGRT on-line corrections; on-board Kv CBCT, acquiring volumetric images inside the treatment room, allows daily detection and correction of systematic and random errors before each treatment session, with a small-dose exposure and good image quality [14-17].

## Conclusions

In our analysis stereotactic fractionated radiotherapy have the significant limitation of uncertainty in inter-fraction repeatability of the patient set-up; IGRT using cone-beam computed tomography improves the accuracy of the treatment delivery reducing set-up uncertainty, giving the possibility of 3-dimensional anatomic informations in the treatment position.

## Competing interests

The authors declare that they have no competing interests.

## Authors' contributions

GI analyzed data and drafted the manuscript; RM analyzed data and drafted the manuscript; DF collected and analyzed data; EP collected and analyzed data; MB collected data; RB revised literature; LDM revised literature; EG collected data; FS collected data; RS revised the manuscript. All authors read and approved the final manuscript.
